# Pediatric cardiac services in Iraq: current status and future plans

**DOI:** 10.1186/1749-8090-8-S1-O303

**Published:** 2013-09-11

**Authors:** A Almandel, H Younis, C Gilbert, S Marr, K Bowtell, B Forsberg, P Shauchenka, E Suslin, F Molloy, W Novick

**Affiliations:** 1 Cardiac Surgery, Nasiriyah Heart Center, Nasiriyah, Iraq; 2 International Children's Heart Foundation, Memphis, TN, USA; 3 Department of Surgery, University of Tennessee Health Sciences Center and International Children's Heart Foundation, Memphis, TN, USA

## Background

Pediatric cardiac services in Iraq are poorly developed. Only two centers in the country provided pediatric cardiac services at any significant level prior to an assistance development plan. Prior to 2010 approximately 400 children/year received surgical care in Iraq. Occasional children were sent out of the country for surgery. We instituted a multi-city assistance program in 2010 and report the results.

## Methods

The database of the International Children’s Heart Foundation was searched for all patients receiving surgery in Iraq. The sites of assistance were Sulimaniyah, Nasiriyah, Najaf and Basra in the order in which assistance was started. Two week mission trips were performed in all sites, but a program of near continuous presence was instituted in Nasiriyah in July 2012. Total and specific institutional analysis was performed. The RACHS-1 model of risk classification was employed for analysis.

## Results

Total mission trips were 14 (Nasiriyah 6, Najaf 5, Sulimaniyah 2, Basra 1) and beginning in August 2012 until May 31, 2012 we have spent 33 weeks in-country. The total number of patients undergoing primary operations was 425. The distribution of cases and mortality by RACHS-1 was; 1- 51/425 (14%) and 1.9% (1/51); 2- 179/425 (48%) and 3.4% (6/179); 3- 122/425 (33%) and 16% (20/122); 4- 17/425 (6%) and 59% (10/17); there was only one RACHS 6 operation. There were 49/425 adults and 6/425 non-classifiable pediatric cases. Mortality and total case volume improved over time p < 0.05 mortality and p< 0.01 for case volume. Neonates comprised 4% of the total cases with mortality of 18.7%.

## Conclusions

Pediatric cardiac surgery in Iraq has increased over time with our assistance. Neonatal cardiac surgery is now offered for the first time in Iraq.

